# Temperature Peak-Drift Correction Method for NaI(Tl) Detectors Using the Background Peak

**DOI:** 10.3390/s24082621

**Published:** 2024-04-19

**Authors:** Songlin Wen, Rong Zhou

**Affiliations:** College of Physics, Sichuan University, Chengdu 610065, China; wensonglin@stu.scu.edu.cn

**Keywords:** NaI(Tl) detector, temperature peak drift, the background peak

## Abstract

The overall gain of a scintillation detector is temperature-dependent, leading to a drift in the measured gamma energy spectrum with changes in temperature. To mitigate this effect, a temperature drift correction is essential prior to conducting gamma spectrum analysis. In this study, the detector gain ratio is determined by comparing the positions of the same background peak across different spectra. Subsequently, the original spectrum is adjusted accordingly to obtain a gamma spectrum free from temperature drift. Experimental results demonstrate that after implementing this correction, the relative deviation of the ^57^*Co* characteristic peak positions in the gamma spectrum measured by the NaI(Tl) detector is reduced from 18.64% to 0.91%. Furthermore, by performing energy calibration beforehand, the characteristic peak position can be utilized for secondary correction, further minimizing temperature drift. Our findings indicate that the relative deviation of the ^22^*Na* characteristic peak positions was reduced, respectively, to 0.51% and 0.46% through secondary correction. This approach, which utilizes the background peak for correction, avoids the need for additional radioactivity or circuitry and effectively mitigates peak drift. Overall, this method holds significant implications for enhancing the accuracy of gamma spectrum analysis.

## 1. Introduction

Scintillation radiation detectors, recognized for their remarkable detection efficiency in nuclear radiation and capacity to measure energy spectra, have found widespread applications across various fields, including environmental research, nuclear medicine, experimental nuclear physics, and high-energy physics [1,2]. However, outside laboratory environments, these detectors experience fluctuations in overall gain due to factors such as the temperature dependency of light output, decay time constant of scintillation, temperature drift of the Photomultiplier tube (PMT), and temperature behavior of associated electronic components [3,4,5,6]. These fluctuations compromise the stability of the energy-channel relationship in gamma spectra, leading to noticeable peak shifts and significant errors in qualitative and quantitative analyses.

Several methods have been developed to mitigate spectral drift. One approach involves minimizing temperature changes directly. For instance, in the COSINE-100 experiment, a crystal array was submerged in 2200 L of scintillation fluid to maintain the temperature near the crystal within ±0.1 °C, effectively eliminating the temperature-induced peak drift in the measured gamma spectrum [7]. However, the impractical size of this setup limits its use to specific locations.

Another method involves correcting the PMT’s anode output waveform through signal processing. Modifications to subsequent circuits allow for the elimination of temperature-induced differences between radiation pulses through pulse deconvolution, trapezoidal shaping, and amplitude correction [8]. Additionally, adjusting the integration time to 385 ns instead of 1.23 µs mitigates temperature-dependent energy calculation, reducing spectral shift [9]. Nevertheless, these methods can introduce complexity to the electronics system and only address temperature drift in scintillation and PMT, neglecting other factors influencing PMT gain and temperature effects on subsequent circuits.

A more direct approach involves compensating for gain based on real-time temperature during measurement and a predetermined relationship. This involves obtaining the scintillator light decay time and calculating crystal temperature [10] or directly measuring the ambient temperature [11,12]. Compensation is then made based on the temperature-gain correspondence to minimize gain differences between spectra. However, not all detectors facilitate measurements at various temperatures for establishing this relationship, and hysteresis in temperature’s effect on the system gain impedes the online gain stabilization.

The most common approach is transforming the measured gamma spectrum according to the peak position of a known reference peak to eliminate temperature-induced peak position drift. This method eliminates the need for predetermined gain-temperature correspondence. Reference peaks can be sourced from LED [13,14] or standard radioactive sources [15,16,17], but considerations regarding signal stability, safety hazards, and degraded detector sensitivity must be addressed. Alternatively, reference peaks can be derived from radioisotope characteristic peaks in the environment or scintillation crystals [18,19], but obtaining significant reference peaks requires substantial scintillator volume or extended measurement times during which temperature may fluctuate.

During ambient background gamma spectroscopy using a NaI(Tl) detector, we identified a significant asymmetric peak in the low-energy range suitable as a reference peak. Consequently, this paper proposes a method to redistribute the gamma spectrum based on the current background peak position to mitigate temperature-induced offsets.

## 2. Theory and Methods

### 2.1. The Energy Calibration Equation

Gamma-ray detection in a scintillation detector involves several crucial processes: (1) Incident gamma rays deposit energy in the crystal through physical processes such as photoelectric absorption, Compton scattering, and electron pair effects; (2) The crystal subsequently emits fluorescence upon de-excitation; (3) Light collection and photoelectron generation occur at the PMT photocathode; (4) Photoelectrons undergo multiplication on PMT dynodes; (5) The pulse is amplified and shaped in the subsequent readout circuit; (6) Finally, the pulse undergoes analog-to-digital conversion and multichannel analysis by the MCA (multichannel analyzer).

Broadly speaking, the overall gain of the detector is influenced by factors including the light output of the scintillation, the multiplication factor of PMT, and the amplification of the electrical signal in the readout electronics system. Temperature variations affect these components, resulting in an inconsistent relationship between the deposited energy *E* and the corresponding channel ch at different temperatures, a phenomenon described by the Energy Calibration Equation as

(1) $$E=a\cdot ch^{2}+b\cdot ch+c,$$

where *a* represents the nonlinear gain coefficient of the detector, *b* is the linear amplification, and *c* represents the energy corresponding to when the channel is zero.

Typically, the measured gamma-ray energy falls within the range of 100 keV to 3 MeV, where the overall gain of the scintillation detector demonstrates satisfactory linearity, rendering the nonlinear gain coefficient *a* negligible. Consequently, the Energy Calibration Equation can be simplified accordingly.

The detection system translates the same energy distribution into different pulse amplitude distributions, leading to temperature-induced peak drift in the gamma spectrum. However, when multiple characteristic peaks are present in the energy spectrum, the Energy Calibration Equation can be derived from the position and the corresponding energy of these peaks. These peaks typically originate from external standard radioactive sources or radioactive isotopes in the environment and scintillation materials, allowing for known energy values to be assigned to each peak.

### 2.2. The Gaussian Fitting

The position of each peak can be calculated based on the number of photoelectrons generated by the PMT photocathode following gamma-ray energy deposition. This process yields discrete carrier numbers due to the inherent statistical fluctuation in the total number of photoelectrons produced, described by a Poisson distribution. Assuming no other causes for the variation in signal amplitude, the detector’s response function follows a Gaussian distribution.

When multiple gamma rays of the same energy deposit all their energy in the detector, a Gaussian-shaped peak emerges in the spectrum of pulse amplitude distribution, often termed the full-energy peak or characteristic peak. Therefore, the precise position of the characteristic peak can be determined by least-squares fitting of the peak-area data using a specific form of the Gaussian function as

(2) $$f\left(x\right)=d_{1}\cdot exp\left(-{\left(x-d_{3}\right)}^{2}/d_{2}^{2}\right)+d_{4},$$

where $d_{1}$ represents the peak count after subtracting the background count, $d_{2}$ represents the standard deviation of the peak position, with the Full Width at Half Maximum (FWHM) of the peak equal to 2.355 times $d_{2}$; $d_{3}$ denotes the value of the full-energy peak position, and $d_{4}$ denotes the count due to Compton scattering of higher energy rays and background radiation from the ambient.

### 2.3. The Correction Process

Using the energy calibration relationship established for each spectrum, it becomes feasible to make modifications. Consider two energy spectra measured at different temperatures: one serves as the standard spectrum, while the other, termed the drift spectrum, requires correction. Initially, when multiple characteristic peaks are present in the energy spectrum, the Energy Calibration Equation can be derived from the position and corresponding energy of these peaks with the position determined through Gaussian fitting and the energy known. The energy range corresponding to any channel *i* in the standard spectrum is computed, and the upper and lower limits of the channel interval corresponding to this range are utilized for integration in the drift spectrum. The resulting integral value represents the count of channel *i* and through iterative calculation, a new pulse amplitude distribution is derived from the drift spectrum after conversion maintaining the same gamma-ray energy distribution information. This new pulse amplitude distribution is denoted as the corrected spectrum, ensuring the elimination of temperature drift through the utilization of the Energy Calibration Equation for the standard spectrum.

Due to the quantization inherent in the analog-to-digital conversion process, the pulse amplitude distribution appears discrete. Assuming that counts within a channel conform to a uniform distribution, the correction process outlined above can be expressed as

(3) $${Correct}_{i}=\int_{\frac{b_{s}\cdot \left(i-1\right)+c_{s}-c_{d}}{b_{d}}}^{\frac{b_{s}\cdot \left(i\right)+c_{s}-c_{d}}{b_{d}}}{Drift}_{ch}\;dch,\;i=1,\ldots ,1024.$$

In the formula, ${Correct}_{i}$ is the count value corresponding to channel *i* in the corrected spectrum, where *i* is an integer. ${Drift}_{ch}$ is the count value corresponding to channel ch in the drift spectrum, and ch can be a decimal. In the equation, $b_{s}$ and $c_{s}$ are the energy calibration parameters of the standard spectrum, while $b_{d}$ and $c_{d}$ represent the parameters of the drift spectrum. Moreover, *n* is the total number of channels in the MCA, and it is also the maximum value taken by *i*.

### 2.4. The Simplified Correction

**Figure 1:**
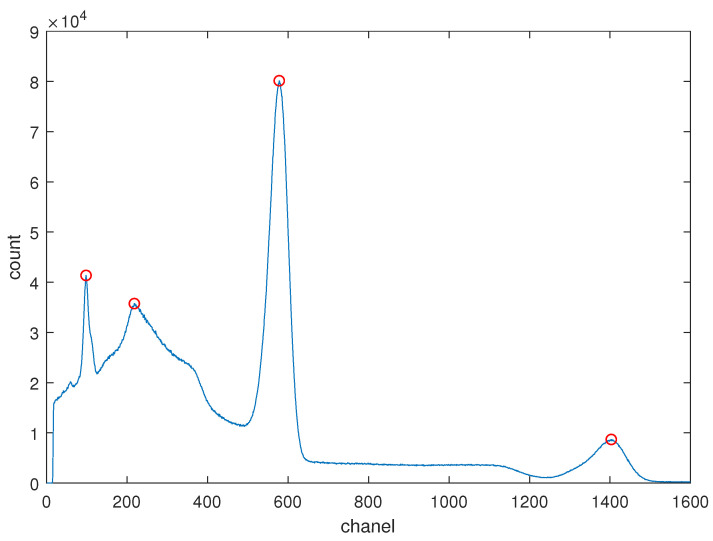
The gamma spectrum of a high-activity ^22^*Na* radioactive source. Red circles are used to mark peaks.

Furthermore, the zero-channel energy *c* remains nearly constant when only the temperature is varied, while other measurement conditions of the detector remain constant. To substantiate this assertion, gamma spectra from a high-activity ^22^*Na* radioactive source were recorded using a NaI(Tl) detector at various temperatures. Figure 1 illustrates the gamma spectrum of the ^22^*Na* source, showcasing four distinct peaks: the backscattering peak, the background peak, and the two characteristic peaks at energies of 511 keV and 1274 keV, respectively.

Energy calibration was conducted using the peak positions and energies of these characteristic peaks, and the corresponding linear amplification factor *b* and the zero-channel energy *c* were calculated at different temperatures, as summarized in Table 1.

The experimental results indicate that the variation in the value of the zero-channel energy *c* at different temperatures is minimal compared to the linear amplification factor *b*. Consequently, *c* can be treated as a constant. Therefore, the correction only requires the ratio of the linear amplifications $b_{s}$ to $b_{d}$ and Equation (3) is rewritten as

(4) $${Correct}_{i}=\int_{\frac{b_{s}\cdot \left(i-1\right)}{b_{d}}}^{\frac{b_{s}\cdot \left(i\right)}{b_{d}}}{Drift}_{ch}\;dch,\;i=1,\ldots ,1024.$$

### 2.5. Correction with the Background Peak

In nature, background radiation, originating from sources like radon gas, radioactive minerals in soil and rocks, fallout from nuclear tests, and cosmic rays interacting with the atmosphere perpetually exists at low levels. This background radiation contributes to gamma spectrum counts even in the absence of a radioactive source near the detector. Notably, our study identified a distinct asymmetric peak in the low-energy segment of gamma spectra when measuring background radiation or radioactive sources. Termed the background peak, it arises from background radiation.

**Figure 2:**
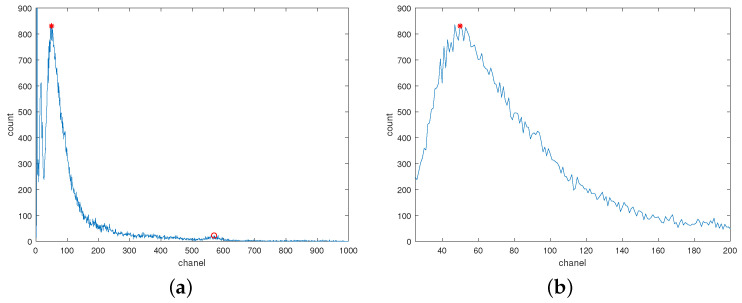
The background spectrum. Red asterisks are used to mark the background peak and red circles are used to mark the peak of K-40. (**a**) The complete background spectrum; (**b**) The shape of the background peak.

The characteristic peaks formed by radioactive isotopes in the environment are often subtle due to their low content, leading to potential errors in peak position calculation. As illustrated in Figure 2a, the background peak is more pronounced than the characteristic peak of K40, facilitating accurate peak position determination through Gaussian fitting.

Furthermore, as depicted in Figure 2b, the count on the right side of the background peak tends to decrease with increasing channel numbers. This asymmetry arises from different factors influencing each side: gamma rays with higher energy have lower interaction probabilities, while rays with extremely low energy are absorbed by the detector shell, resulting in a sharp count drop below a certain threshold. Thus, the background peak’s position corresponds to the energy determined by the detector shell, enabling its use in temperature drift correction.

Given that the zero-channel energy *c* remains constant regardless of temperature variations, differences in pulse amplitude distribution arise solely from the linear amplification *b*. Background radiation measurement occurs under a specific temperature, yielding the standard spectrum, while spectra obtained at other temperatures serve as drift spectra. Using the channel positions of the background peak in both standard and drift spectra, the linear amplification ratio before and after temperature changes (*k*) can be determined as

(5) $$k=\frac{b_{s}}{b_{d}}=\frac{ch_{s}}{ch_{d}},$$

where $b_{s}$ and $b_{d}$ represent the detector’s linear amplification at room temperature and after a temperature change, respectively, while $ch_{s}$ and $ch_{d}$ denote the channel positions of the background peak in the standard and drift spectra, respectively.

**Figure 3:**
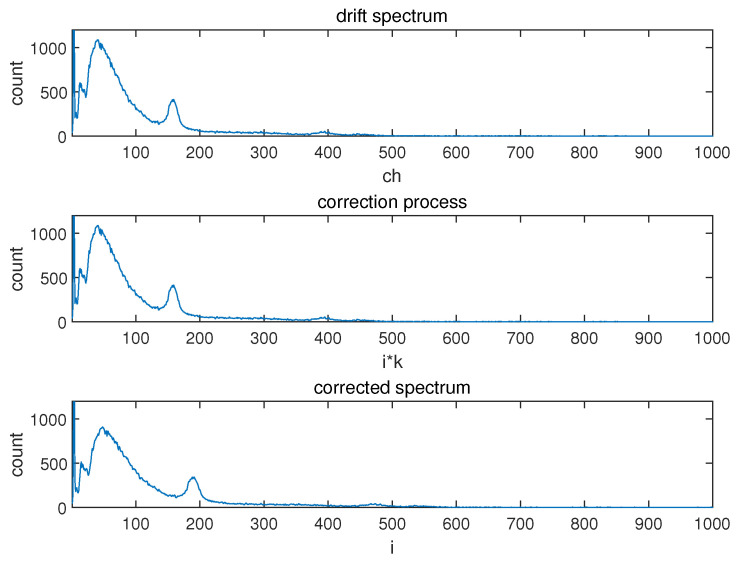
The process of converting from a drift spectrum to a corrected spectrum.

Figure 3 illustrates the graphical depiction of the entire correction process, which involves stretching or compressing the original distribution on the horizontal axis based on the background peak position ratio between the drift and standard spectra (*k*).

## 3. Results

### 3.1. The Experimental Setup

The experimental data presented in this paper were gathered using a NaI(Tl) detector featuring a 2-inch by 2-inch NaI(Tl) crystal coupled with a Hamamatsu CR105-02 photomultiplier tube (PMT). The PMT anode’s output signal undergoes amplification and reshaping in the readout electronics system before being fed into the interface of an STM32 microcontroller. The microcontroller handles the analog-to-digital conversion of signals and records their maximum amplitude value. Utilizing a charge-sensitive preamplifier, the maximum amplitude of output signals reflects the deposited energy, serving as a physical quantity for channel calculation. A complete pulse amplitude distribution is obtained at the conclusion of the measurement period.

**Figure 4:**
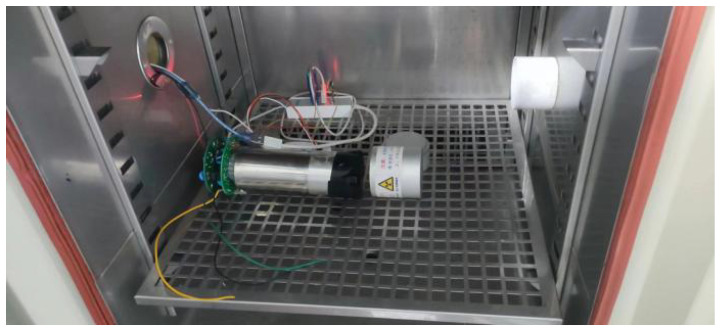
The experimental device.

To evaluate the practical effectiveness of the correction method proposed in this study, an experimental setup was devised as illustrated in Figure 4. The entire detector assembly was housed within a temperature-adjustable thermostat, with a standard radioactive source positioned in front of the detector for measurement purposes.

The thermostat’s temperature was set to span the operating temperature range of the detector, ranging from −20 °C to 50 °C with a step size of 10 °C. Each temperature point was maintained for 30 min to ensure internal thermal equilibrium, confirmed by monitoring the thermostat’s display. Subsequently, the detector commenced gamma spectrum measurements, with each measurement lasting 600 s.

### 3.2. The Correction of ^57^*Co* Source

**Figure 5:**
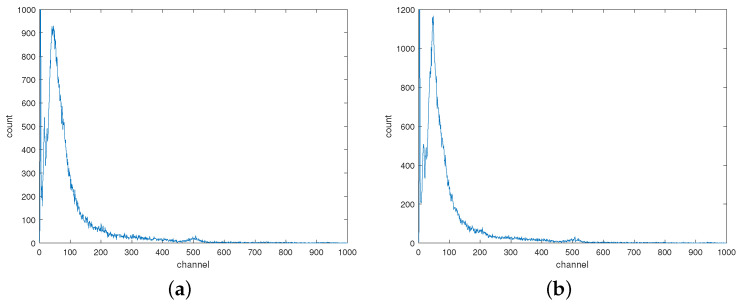
The gamma spectra at room temperature. (**a**) The background radiation spectrum; (**b**) The gamma spectrum of ^57^*Co*.

A low-activity ^57^*Co* radioactive source was chosen as the detection target, and both the background radiation spectrum and the gamma spectrum of ^57^*Co* were measured at room temperature. These spectra are depicted in Figure 5a and Figure 5b, respectively, with channel plotted on the horizontal axis and count on the vertical axis. Comparing the two spectra, the primary difference lies in the larger counts of the background peak in the gamma spectrum of ^57^*Co*. This discrepancy arises because the characteristic peaks of ^57^*Co* are closely positioned to the background peak, and the energy resolution of the NaI(Tl) detector does not adequately differentiate between the two. Therefore, the gamma spectra of ^57^*Co* at various temperatures serve as a typical example for testing the effectiveness of the method in correcting characteristic peaks with low energy and proximity to the background peak.

**Figure 6:**
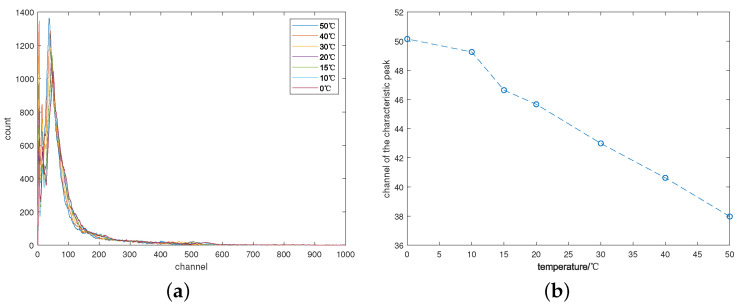
The uncorrected gamma spectra of ^57^*Co*. (**a**) The gamma spectra of ^57^*Co* at different temperatures; (**b**) Curves for the characteristic peak position with temperature changes.

After measuring the gamma spectra of ^57^*Co* at different temperatures, a series of uncorrected gamma spectra were plotted in Figure 6a, while the positions of the same characteristic peak at varying temperatures were plotted in Figure 6b. Significant drift is observed between the different gamma spectra, with the characteristic peak position gradually shifting leftward as the temperature rises, following a pattern similar to the change in light yield.

**Figure 7:**
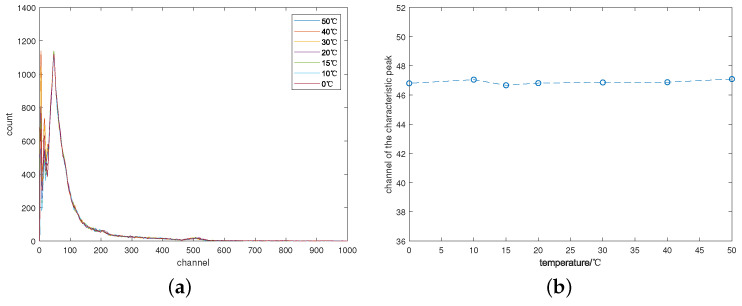
The corrected gamma spectra of ^57^*Co*. (**a**) The gamma spectra of ^57^*Co* at different temperature; (**b**) Curves for the characteristic peak position with temperature changes.

Given that the room temperature during measurement was 15 °C, the background radiation spectrum measured at this temperature was chosen as the standard spectrum. The ratio of the background peak position in the standard spectrum and each gamma spectrum was calculated separately, and the drift spectrum was corrected accordingly. The corrected gamma spectra and the positions of the same characteristic peaks were then plotted in Figure 7a and Figure 7b, respectively, following the same conventions as before. Notably, all spectra exhibit excellent overlap, demonstrating that correction based on background peaks effectively eliminates temperature drift in the gamma spectrum measured by the NaI(Tl) detector.

To further illustrate the corrective effect of the method, Gaussian fitting was employed to determine the precise position of the same characteristic peaks in the drift spectra and the corrected spectra, respectively. Subsequently, the relative deviation of the positions is presented in Table 2. The formula for calculating this value is

(6) $$RD=\frac{\left(ch_{j}-ch_{t}\right)}{ch_{t}}\cdot 100\%,$$

where RD is the relative deviation of peak positions, $ch_{t}$ is the characteristic peak position in the spectrum measured at 15 °C and $ch_{j}$ is the characteristic peak position in any spectrum measured at other temperatures.

Overall, the method effectively corrects temperature drift in gamma spectra, as evidenced by the improved alignment of the characteristic peak positions across varying temperatures.

### 3.3. The Correction of ^22^*Na* Source

#### 3.3.1. Initially Correction

**Figure 8:**
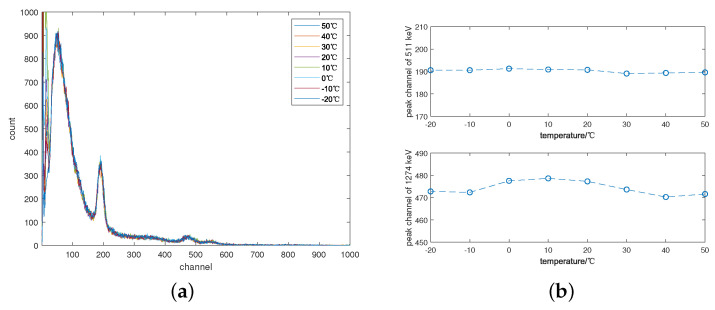
The corrected gamma spectra of ^22^*Na*. (**a**) The gamma spectra of ^22^*Na* at different temperatures; (**b**) Curves for the characteristic peak position with temperature changes.

For the correction, the background radiation spectrum measured at 20 °C is selected as the standard spectrum due to the ease of reaching this temperature condition. Similar to the ^57^*Co* source discussed earlier, a low-activity ^22^*Na* source is chosen as the detection target. The gamma spectra of ^22^*Na* measured at various temperatures are designated as the drift spectra and corrected utilizing the background peak method. Subsequently, the corrected gamma spectra and the positions of the corresponding characteristic peaks are plotted in Figure 8a and Figure 8b, respectively. Notably, the method demonstrates significant correction effectiveness for characteristic peaks with energies around 1 MeV.

#### 3.3.2. Secondary Correction

In the simplified correction method discussed earlier, only the background peak is utilized as a reference peak, resulting in a simpler process compared to other methods. However, slight differences in the Energy Calibration Equation of the corrected spectra may arise due to the omission of the change in the zero-energy channel *c*. To further reduce temperature drift, a secondary correction method is employed utilizing the position of the characteristic peak formed by the detection target in the corrected spectrum.

To initiate the secondary correction, it is crucial to determine the energy corresponding to the characteristic peak. This is typically accomplished by measuring the gamma spectra of known radioactive sources under the same temperature as the standard spectrum, facilitating the derivation of the Energy Calibration Equation. Utilizing this equation allows for the calculation of an approximation of the energy and consulting a nuclear database provides a reliable method to obtain the true value. In the present experiment, the detection target is a ^22^*Na* source, with characteristic peaks of 511 keV and 1274 keV evident in every measured gamma spectrum. Given this knowledge, the calibration, calculation, and consultation process can be expedited or skipped altogether.

Subsequently, the energies and positions of the characteristic peaks of the target are used to calculate the linear amplification *b* and the zero-energy-channel *c* for each spectrum. The unaltered correction described in Equation (3) is then applied to the corrected spectra, yielding the final pulse amplitude distribution recorded as the secondary corrected spectrum.

Table 3 illustrates the significant increase in peak drift and relative deviation observed across various temperatures before correction. Over the temperature range of −20 °C to 50 °C, the relative deviation of the 511 keV peak between the spectrum measured at 20 °C and others fluctuated by up to 19.39%, while for the 1274 keV peak, it reached a maximum of 19.88%. However, after correction using only background peaks, both deviations improved substantially, with new maximum relative deviations of 0.94% and 1.53%, respectively. Further correction of the corrected spectrum using the characteristic peaks of ^22^*Na* resulted in even lower values of 0.51% and 0.46%, respectively.

## 4. Discussion

Compared to existing methods, utilizing the background peak to correct temperature-induced peak drift in NaI(Tl) detectors offers several advantages.

Firstly, from a cost perspective, the correction process involves only extracting the position of the background peak in the measured gamma spectrum without incurring additional material or management costs. Unlike conventional energy spectrum measurements, this method does not require acquiring extra information, eliminating the need for circuit modifications or additional electronic components. Moreover, since the background peak is generated by background radiation, there is no necessity for external standard radiation sources or LED light sources to obtain reference peaks.

In the correction process, obtaining the position of the background peak through Gaussian function fitting in the specified region for each spectrum, and subsequently stretching or compressing the drift spectrum on the horizontal axis based on the ratio of the background peak position are simple tasks feasible to be performed on a single-chip computer. This allows for direct transmission of the corrected spectrum from the detector to the computer.

The background peak, unlike the characteristic peak formed by radiation of a specific energy, arises from the varied probabilities of gamma rays with different energies interacting with the scintillation. It encompasses a broader range of sources and possesses a more pronounced appearance compared to the characteristic peaks caused by radioisotopes in the environment. Consequently, this method necessitates a relatively shorter measurement time and mitigates the impact of significant temperature variations during the measurement process.

Crucially, experimental results demonstrate the effectiveness of this method in eliminating peak drift caused by temperature changes in detectors. For instance, in the gamma spectrum of ^57^*Co*, the relative deviation of corrected characteristic peak positions decreased from 18.64% to 0.91%. Furthermore, by determining the energies corresponding to characteristic peaks in the calibrated spectrum using the energy calibration equation of the standard spectrum, the type of radioisotope in the target under test can be identified. Secondary calibration, based on multiple reference peaks in the calibration spectrum, can yield even better results. For instance, in the gamma spectrum of ^22^*Na*, the relative deviations of two characteristic peak positions decreased from 19.39% and 19.88% to 0.51% and 0.46%, respectively, after secondary calibration.

In other aspects, since no external radioactive source is used, there is no risk of loss, and operators are not exposed to additional radiation. At the same time, the sensitivity and accuracy of NaI(Tl) detectors are not compromised by the incoming rays used in the correction process. In addition, the reference peak used in correction typically exhibits larger fluctuations, whereas the background peak, owing to its low energy, has a smaller range, making it easier to locate.

## 5. Conclusions

In this paper, we propose that the peak drift observed in gamma spectra is a consequence of changes in the overall gain of detectors at different temperatures. While theoretically the current detector gain can be determined by deriving the relationship between deposited energy and channel using reference peaks in spectra, practical measurements may lack characteristic peaks suitable for energy calibration. To address this challenge, we utilize the background peak as the reference point and obtain the ratio of linear gain by comparing its position in the drift spectrum and the standard spectrum. By adjusting the original pulse amplitude distribution based on the ratio, we effectively eliminate temperature-induced peak drift. Subsequently, the energy of the target characteristic peak can be determined through approximation and consulting nuclear databases, serving as a reference point for further correction.

Overall, the correction of peak drift using the background peak emerges as an efficient method for reducing errors in both qualitative and quantitative analyses across various temperatures. Although the method is demonstrated specifically for NaI(Tl) detectors in this paper, its theoretical applicability extends to other scintillator detectors as well.

## Figures and Tables

**Table 1:** The characteristic peak positions and energy calibration relationship parameters at different temperatures.

Temperature/°C		50	40	30	20	10	0	−10	−20
Peak	511 keV	548.72	568.08	578.25	576.11	559.01	521.89	468.57	435.65
Positions	1274 keV	1327.84	1374.89	1399.42	1394.36	1352.61	1262.81	1133.15	1054.39
Energy	*b*	0.98	0.95	0.93	0.93	0.96	1.03	1.15	1.23
Calibration	*c*	−26.36	−26.23	−26.28	−26.21	−26.45	−26.45	−26.95	−26.24

**Table 2:** The characteristic peak position and relative deviation of ^57^*Co* before and after initial correction.

Temperature/°C		50	40	30	20	15	10	0
Drift Spectrum	Position	37.97	40.63	42.99	45.67	46.67	49.26	50.15
	RD	18.64%	12.95%	7.89%	2.14%	0.00%	5.56%	7.47%
Corrected Spectrum	Position	47.10	46.88	46.86	46.82	46.67	47.05	46.80
	RD	0.91%	0.45%	0.41%	0.32%	0.00%	0.82%	0.29%

**Table 3:** The peak position and relative deviation of ^22^*Na* characteristic peaks before and after correction.

Temperature/°C			50	40	30	20	10	0	−10	−20
Drift Spectrum	511 keV	Position	157.74	165.32	177.12	195.68	201.56	211.18	215.30	210.30
		RD	19.39%	15.52%	9.49%	0.00%	3.00%	7.92%	10.03%	7.47%
	1274 keV	Position	392.51	410.87	442.63	489.92	505.14	528.33	534.35	522.93
		RD	19.88%	16.14%	9.65%	0.00%	3.11%	7.84%	9.07%	6.74%
Corrected Spectrum	511 keV	Position	189.48	188.80	188.80	190.59	190.61	191.20	190.71	190.44
		RD	0.59%	0.94%	0.94%	0.00%	0.01%	0.32%	0.06%	0.08%
	1274 keV	Position	471.50	469.76	473.38	477.08	478.36	477.24	472.36	472.39
		RD	1.17%	1.53%	0.77%	0.00%	0.27%	0.03%	0.99%	0.98%
Secondary Corrected	511 keV	Position	196.13	196.92	197.02	196.44	196.02	195.64	195.43	196.04
		RD	0.16%	0.24%	0.30%	0.00%	0.21%	0.41%	0.51%	0.20%
	1274 keV	Position	491.82	494.11	491.89	493.82	493.68	491.57	493.42	492.24
		RD	0.41%	0.06%	0.39%	0.00%	0.03%	0.46%	0.08%	0.32%

## Data Availability

The data that support the findings of this study are available from the corresponding author upon reasonable request.
